# Comprehensive Characterization of the Regulatory Landscape of Adrenocortical Carcinoma: Novel Transcription Factors and Targets Associated with Prognosis

**DOI:** 10.3390/cancers14215279

**Published:** 2022-10-27

**Authors:** João C. D. Muzzi, Jéssica M. Magno, Jean S. Souza, Larissa M. Alvarenga, Juliana F. de Moura, Bonald C. Figueiredo, Mauro A. A. Castro

**Affiliations:** 1 Laboratório de Imunoquímica (LIMQ), Pós-Graduação em Microbiologia, Parasitologia e Patologia, Departamento de Patologia Básica, Universidade Federal do Paraná (UFPR), Curitiba 81530-990, Brazil; 2 Laboratório de Bioinformática e Biologia de Sistemas, Pós-Graduação em Bioinformática, Universidade Federal do Paraná (UFPR), Curitiba 81520-260, Brazil; 3 Oncology Division, Instituto de Pesquisa Pelé Pequeno Príncipe, Curitiba 80250-060, Brazil; 4 Molecular Oncology Laboratory, Centro de Genética Molecular e Pesquisa do Câncer em Crianças (CEGEMPAC), Curitiba 80030-110, Brazil

**Keywords:** adrenocortical carcinoma, transcriptomics, regulatory network, transcription factor, immuno-oncology, KI-67, *NR5A1*, *CENPA*, *ZBTB4*, *IZKF1*

## Abstract

**Simple Summary:**

Adult adrenocortical carcinoma (ACC) is a rare and aggressive tumor in adults, usually associated with excessive steroid secretion. It is highly metastatic and has few therapeutic options and a poor prognosis. Here, we explore the hallmarks of ACC influenced by transcription factors and their target genes (regulons) to provide a prognostic overview of ACC biology. Using an in silico clinical data analysis approach, we assessed human transcriptomic data from publicly available datasets. We found four distinct clusters of regulons associated with good and worse prognoses associated with cell proliferation and/or immunologic activity. Some findings require further bench analyses, primarily focusing on worse prognostic regulons and their targets.

**Abstract:**

We reconstructed a transcriptional regulatory network for adrenocortical carcinoma (ACC) using transcriptomic and clinical data from The Cancer Genome Atlas (TCGA)-ACC cohort. We investigated the association of transcriptional regulatory units (regulons) with overall survival, molecular phenotypes, and immune signatures. We annotated the ACC regulons with cancer hallmarks and assessed single sample regulon activities in the European Network for the Study of Adrenal Tumors (ENSAT) cohort. We found 369 regulons associated with overall survival and subdivided them into four clusters: RC1 and RC2, associated with good prognosis, and RC3 and RC4, associated with worse outcomes. The RC1 and RC3 regulons were highly correlated with the ‘Steroid Phenotype,’ while the RC2 and RC4 regulons were highly correlated with a molecular proliferation signature. We selected two regulons, *NR5A1* (steroidogenic factor 1, SF-1) and *CENPA* (Centromeric Protein A), that were consistently associated with overall survival for further downstream analyses. The *CENPA* regulon was the primary regulator of *MKI-67* (a marker of proliferation KI-67), while the *NR5A1* regulon is a well-described transcription factor (TF) in ACC tumorigenesis. We also found that the *ZBTB4* (Zinc finger and BTB domain-containing protein 4) regulon, which is negatively associated with *CENPA* in our transcriptional regulatory network, is also a druggable anti-tumorigenic TF. We anticipate that the ACC regulons may be used as a reference for further investigations concerning the complex molecular interactions in ACC tumors.

## 1. Introduction

Adrenocortical carcinoma (ACC) is a rare, aggressive endocrine malignancy with a bimodal age distribution and distinct characteristics between pediatric and adult tumors [1,2]. ACC is characterized by a highly proliferative and immune-suppressed tumor microenvironment, high production of corticoids, *TP53* mutation, and an upregulation of the WNT/β-Catenin pathway [3,4,5,6].

Zheng et al. (2016) classified The Cancer Genome Atlas (TCGA)-ACC patients into four groups based on mRNA K4 clustering: Steroid Phenotype High (HSP), HSP + Proliferation, Steroid Phenotype Low (LSP), and LSP + Proliferation [7]. The Steroid Phenotype is related to the activation of steroid biosynthesis pathways, while Proliferation was assessed by a proliferation score proposed by Wirapati et al. (2008) [8]. This classification presented a high overlap with the C1A/C1B molecular classification presented by Reynies et al. (2009) [9], being HSP and HSP + Proliferation related to C1A and worse outcomes, and LSP associated with C1B. In our previous study [6], we have shown that LSP and LSP + Proliferation presented a significant presence of immune infiltration compared to the immune-suppressed microenvironment of HSP and HSP + Proliferation. Furthermore, Landwehr et al. (2020) showed that excessive glucocorticoid levels, present in nearly 60% of ACC patients, are related to T cell depletion in the tumor microenvironment [5].

The functional interplay between the tumor and infiltrating immune cells within the tumor microenvironment provides insights into genes associated with the anti-tumor immune response [10,11,12]. The levels and distribution of CD3+ and CD8+ T cell infiltration distinguish four solid tumor phenotypes: hot (or inflamed), altered excluded, altered immunosuppressed, and cold (or non-inflamed) [11,12,13]. ACC is described as an immunologically cold tumor, presenting one of the lowest immune infiltrates among 30 solid cancer types from TCGA [7,14]. However, the amount and efficacy of the immune infiltrate depend on pre-existing low levels of intratumoral steroids [5,6]. This suggests that in order to boost the anti-tumor immune response, it is necessary to eliminate excessive glucocorticoid levels, and that may be the main reason for heterogeneous results in five clinical trials using four different immune checkpoint inhibitors (avelumab, nivolumab, pembrolizumab, and ipilimumab) [15,16,17,18,19].

2022 WHO Classification of Adrenal Cortical Tumors recommends that diagnosticians specify the mitotic count and the nuclear protein Ki-67 (Ki-67) labeling index in all ACCs [20]. A Ki-67 (or *MKi-67*) labeling index relates to proliferation and malignancy, besides being used in the risk stratification and the rationale of adjuvant mitotane therapy [20,21,22]. Steroidogenic Factor 1 (SF1) immunohistochemistry is the most reliable and specific biomarker to confirm the adrenal cortical origin [23]. This transcription factor (TF) is encoded by the Nuclear Receptor Subfamily 5 Group 1 (*NR5A1*) gene, whose overexpression is associated with increased steroid metabolism, proliferation, and a worse outcome [24,25].

Different combinations of regulators and molecular factors may be associated with cancer development. The inference of regulatory networks helps to understand how these factors may be related, converging on cellular mechanisms, which can add to the understanding of the biology of the disease or intervention strategies [26,27,28,29]. To reconstruct a regulatory network, gene expression data can be used to evaluate mutual information between a TF and potential target genes, generating regulatory hubs called regulons [26]. This reverse-engineering method has been successfully applied in other cancer types (e.g., [26,27,29]).

In the present study, we inferred a transcriptional regulatory network for ACC using publicly available transcriptomic and clinical data from the TCGA-ACC cohort [7]. Through multivariate Cox analysis, we found 369 regulons, composed of a TF and its direct and indirect targets, relating to overall survival. We investigated how these regulons correlate with molecular phenotypes and immune signatures [14]. In addition, we annotated these regulons with Molecular Signatures Database (MSigDb) Hallmarks, representing well-defined biological states or processes [30]. Finally, we tested the prognostic value of the regulon activity in the European Network for the Study of Adrenal Tumors (ENSAT) cohort [31].

## 2. Methods

### 2.1. The Cancer Genome Atlas-ACC Data

The TCGA-ACC RNA-seq and clinical data [7] available from the GDC repository were downloaded using the TCGABiolinks package v.2.20.1 in R [32,33,34]. Next, we assessed the curated survival data of TCGA-ACC participants using the Xena Browser [35]. Then, the gene expression matrix was filtered using the AnnotationHub package v.3.0.2 in R [36] for protein-coding genes. Finally, we normalized the raw counts with the variance stabilizing transformation (VST) method from the DESeq2 package v.1.32.0 in R [37], using the Steroid Phenotype classification in the experimental design (see Section 2.6).

### 2.2. Regulon Inference

The normalized gene expression matrix was used to call regulons with the RTN package v.2.16.0 in R [26]. First, we reconstructed regulons for 1605 TFs [38,39] using the ARACNe algorithm [40]. Then, we used the *tni.alpha.adjust*() function [41] to define a statistical threshold that controls the trade-offs between Type I and Type II errors at a similar level described by Castro et al. (2016) [26], using a Benjamini-Hochberg [42] adjusted *p*-value cutoff of 0.05.

### 2.3. Regulon Activity

The regulon activity was estimated using a two-tailed gene set enrichment analysis (GSEA2) [26] which produces a differential enrichment score (dES) for each sample. A positive dES represents activated regulons, while a negative dES represents suppressed regulon activity. Values near zero indicate inconclusive activity. We selected regulons with a minimum of 15 positive and 15 negative targets [30] to assess regulon activity, which is regarded as the minimum gene set size for downstream enrichment analyses [30].

### 2.4. Survival Analysis

We used the regulon activity for multivariate Cox analysis [43] relating to overall survival (OS) and Progression-Free Interval (PFI) (the period from the date of diagnosis until loco-regional or systemic recurrence, second malignancy, or death from any cause) [35]. For this analysis, we considered the tumor stage and age as covariates using the RTNSurvival package v.1.20.0 pipeline [44], generating the hazard ratio (HR) and 95% confidence interval (CI) for each regulon. Since the Cox analysis assessed the time-to-event association between steroid-related regulons and OS, steroid-secreting status was not included in the Cox model due to potential co-linearity with steroid-related regulons. Therefore, the regulons with an adjusted *p*-value below 0.05 for OS were selected for the subsequent analysis. For Kaplan-Meier survival curves [45], samples were divided into high, inconclusive, or low regulon activity, and the *p*-values were calculated using log-rank statistics [46,47].

### 2.5. Clustering

The regulons’ activity dES was used for the unsupervised consensus clustering using the ConsensusClusterPlus package v.1.56.0 in R [48]. We chose k equal to four.

### 2.6. Steroid and Proliferation Classification

Of the 92 samples in the TCGA-ACC cohort, 79 had RNA-seq data, and 78 were listed in the mRNA K4 molecular classification [7], which assigns a Steroid Phenotype to the samples.

The mRNA K4 classified the participants in “steroid-phenotype-high” (*n* = 25), “steroid-phenotype-high + proliferation” (*n* = 22), “steroid-phenotype-low” (*n* = 27), and “steroid-phenotype-low + proliferation” (*n* = 4).

We separated the Steroid Phenotype and the Proliferation profiles, resulting in two groups for the Steroid Phenotype, HSP (*n* = 47) and LSP (*n* = 31), and two groups for the higher and lower proliferation scores (*n* = 26 and 52, respectively). The proliferation score used by Zheng et al. (2016) [7] was based on a proliferation gene set signature described by Wirapati et al. (2008) [8].

### 2.7. Differential Expression for Steroid and Proliferation Phenotypes

We called differentially expressed genes (DEGs) for the Steroid and Proliferation phenotypes using the DESeq2 package v.1.32.0 in R [37]. To avoid confounding effects, the two profiles were independently analyzed, generating DEGs relating to the Steroid independent of Proliferation (IP) classification and the Proliferation independent of Steroid (IS) classification. We considered significant DEGs with adjusted *p*-values below 0.05 in the Wald test [37].

### 2.8. Transcriptional Network Analysis (TNA)

The association between the activity of a regulon and the Steroid and Proliferation phenotypes was assessed using GSEA2 implemented in the TNA pipeline [38]. Here, we used the list of DEGs described in Section 2.7. Associations with adjusted *p*-values below 0.01 were considered significant.

### 2.9. Functional Annotation with MSigDb Hallmarks

The prognostic regulons were annotated with MSigDb Hallmarks [30] using the *tni.annotate.regulons()* function from the RTN package in R [26]. We used the Hallmark gene sets from the msigdbr package v.7.4.1 [49].

### 2.10. Immune Correlation

Values for leukocyte fraction, immune signatures, T-cell receptor (TCR), and B-Cell Receptor (BCR) metrics were retrieved from the master table for TCGA-ACC participants from Thorsson et al. (2018) [14]. We calculated the Spearman correlation between these values and the regulon activity.

### 2.11. Duals Inference

To search for co-regulatory associations between pairs of prognostic regulons, we used the RTNduals pipeline as described in Chagas et al. (2019) [50].

### 2.12. ENSAT Cohort Data

The clinical data and the normalized gene expression matrix were assessed from the ENSAT cohort using the GEOquery package v.2.60.0 [51]. This cohort comprises 44 ACC samples and is available in the Gene Expression Omnibus (GEO) portal under the accession number GSE49278 [31]. When more than one entry from the gene expression data referred to the same gene symbol, we selected the one with the higher coefficient of variation between the samples. The additional clinical data was obtained from the supplementary tables of Assié et al. (2014) [31].

### 2.13. Regulon Activity and Survival Analysis in the ENSAT Cohort

The regulatory network inferred in the TCGA-ACC cohort was used to calculate the regulon activity in the ENSAT cohort using GSEA2 [26]. We selected the regulons related to OS in the TCGA-ACC for multivariate Cox analysis in the ENSAT cohort. The HR and 95% CI for OS were inferred using tumor stage and age as covariates. For the Kaplan-Meier survival analysis, we followed the same protocol described in Section 2.4.

### 2.14. Statistics and Visualization

The R packages available in CRAN [52] and Bioconductor [53] repositories were used for statistical analyses. All *p*-values were corrected for multiple hypotheses using the Benjamini-Hochberg correction [42], and if not specified otherwise, we considered the result significant when below 0.05.

The two-sided Mann-Whitney-Wilcoxon test [54,55] was used for the boxplot comparison when only two pairs were available. For general comparison between more than two groups, we used the Kruskal-Wallis rank-sum test [56] followed by Dunn’s test [57] for multiple pairwise comparisons.

For the construction of heatmaps, we used the ComplexHeatmap package v.2.8.0 [58] in R. To visualize the regulon network, we used the RedeR package v.2.0.0 [59] in R.

## 3. Results

### 3.1. The Identification of 369 Regulons with Prognostic Values Related to Molecular Phenotypes and Leukocyte Fractions

**Figure 1:**
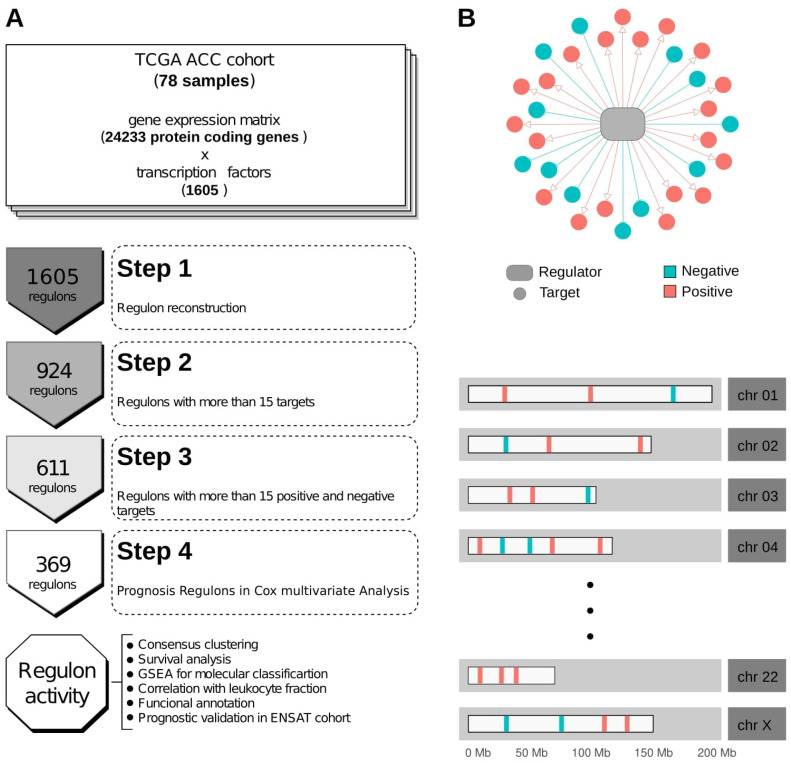
Workflow and regulon representation. (**A**) Schematic workflow of the study. (**B**) Example of a regulon and the distribution of possible target genes in chromosomes.

The TCGA-ACC RNA-seq data was used to call regulons using the RTN R package [26] (Supplementary Table S1). Of the 1605 TFs annotated in the Lambert et al. (2018) collection [39], 611 had at least 15 positive and 15 negative targets in our transcriptional regulatory network (Supplementary Table S2, Supplementary Figure S1), which is regarded as the minimum gene set size for downstream enrichment analyses [30]. In Figure 1A, we present the general workflow used in this study. Figure 1B illustrates an example of a regulon and its targets’ distribution along the karyogram.

**Figure 2:**
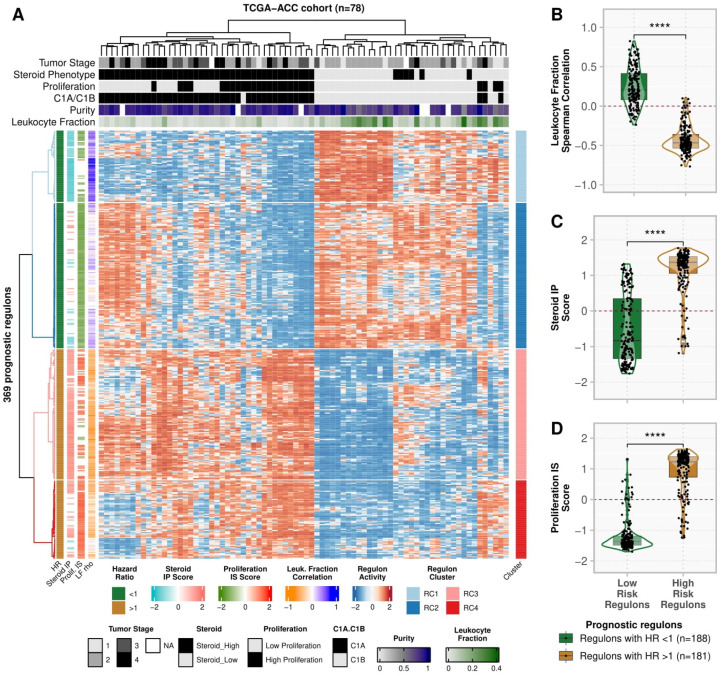
Activity profile of prognostic regulons (*n* = 369) in TCGA-ACC cohort (*n* = 78). (**A**) Heatmap with the activity of the 369 prognostic regulons. Each row represents a regulon, and each column is a sample from the TCGA-ACC cohort. Both were subjected to unsupervised clustering. The upper tracks present the clinical and molecular classification for the samples defined by Zheng et al. (2016) [7] and Thorsson et al. (2018) [14]. The left tracks show the regulon association with the hazard ratio (HR) for overall survival (OS), the Steroid independent of Proliferation (IP) and Proliferation independent of Steroid (IS), and the correlation with the Leukocyte Fraction. The right track presents the regulon clusters defined by Consensus Clustering (Supplementary Figure S3). (**B**–**D**) Regulons are grouped into High- and Low-risk categories according to the HR in Multivariate Cox Analysis. Each point represents a regulon, and the contour presents the distribution density of the regulons for each group. The boxplots show the distinction between the groups for (**B**) the Spearman’s correlation between the leukocyte fraction and the regulon activity, (**C**) the Steroid IP score, and (**D**) the Proliferation IS score. **** *p* ≤ 0.0001 in the two-sided Mann-Whitney-Wilcoxon test.

Using multivariate Cox analysis, we used the OS data to assess the association with the regulons’ activity. Of the 611 regulons, we found 369 related to OS, of which 330 (89.4%) were also related to PFI (Supplementary Tables S3 and S4). Figure 2A shows the activity profile of the 369 prognostic regulons in the TCGA-ACC cohort (Supplementary Table S5). In the unsupervised clustering, the 188 good prognostic regulons showed higher activity in the LSP and C1B participants, and in participants with a higher leukocyte fraction. Conversely, the 181 poor prognosis regulons showed higher activity in the HSP and C1A participants, and participants with a lower leukocyte fraction. Consistently, the activity of high- and low-risk regulons presented opposite correlations with the leukocyte fraction (Figure 2B, Supplementary Table S4) and with the Steroid IP and Proliferation IS scores (Figure 2C and 2D, respectively, Supplementary Tables S4 and S6).

### 3.2. Consensus Clustering Resulted in Four Regulon Clusters with Different Functional and Molecular Characteristics

**Figure 3:**
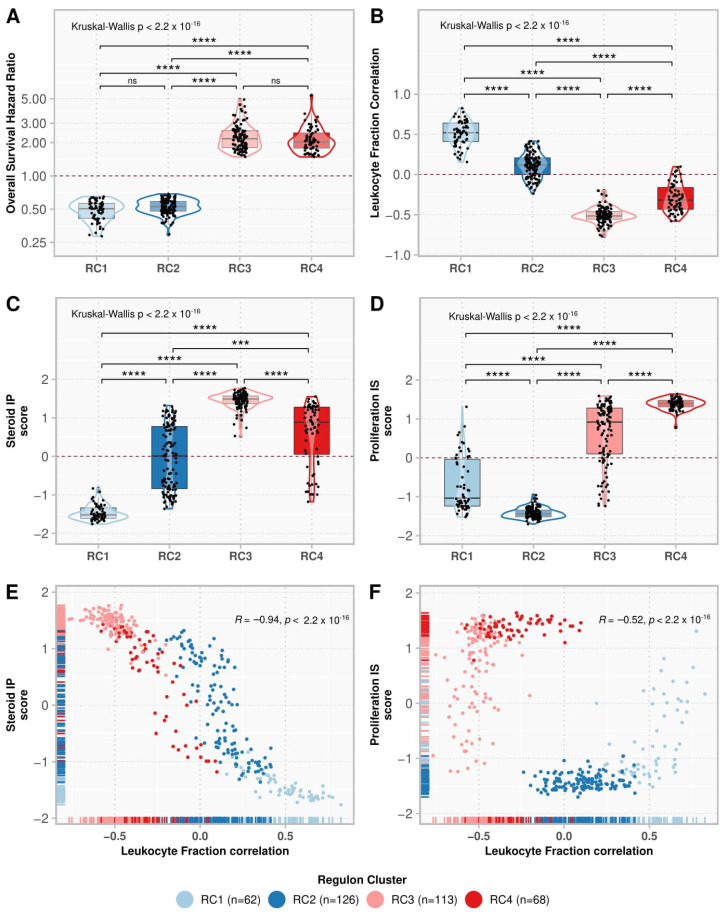
Comparison of regulon clusters (RC). The boxplots show the regulon distributions in each cluster for the overall survival (OS) hazard ratio (HR) (**A**), leukocyte fraction correlation (**B**), Steroid independent of Proliferation (IP) score (**C**), and Proliferation independent of Steroid (IS) score (**D**). Each point represents a regulon, and the box’s horizontal lines show the median and the 25–75% percentiles, while the whiskers (vertical lines) cover the 0–25% and 75–100% percentiles. The contour presents the distribution density of the regulons. The results of Kruskal-Wallis and Dunn’s tests for multiple pairwise comparisons of the ranked data are presented on top. Asterisks indicate the significance level as follows: *** *p* ≤ 0.001, and **** *p* ≤ 0.0001. Non-significant *p*-values (*p* > 0.05) are represented by “ns”. (**E**,**F**) show the scatter plot for the leukocyte fraction correlation with the Steroid IP score and Proliferation IS score, respectively. Each point represents a regulon, colored by its respective cluster. The rugs show the distribution of the points along the *x* and *y*-axes. The Spearman correlation rho and the associated *p*-value are shown in the top-right corner.

We used consensus clustering to look for subgroups within the low- and high-risk regulons (Supplementary Figure S2). The 188 regulons with good prognosis were divided into two clusters: regulon cluster (RC) 1 and RC2, with 62 and 126 regulons, respectively. The 181 regulons related to a worse prognosis were divided into RC3 and RC4, with 113 and 68 regulons, respectively (Figure 3A). RC1 activity showed the highest positive correlation with the presence of immune infiltrate and with the Steroid IP score, as opposed to RC3 (Figure 3B,C). RC2 had the lowest scores for the Proliferation IS classification, in contrast to RC4 (Figure 3D). The regulons’ correlation with leukocyte fraction was negatively associated with the Steroid IP (ρ = −0.94, *p*-value < 2.2 × 10^−16^) (Figure 3E) and the Proliferation IS scores (ρ = −0.52, *p*-value < 2.2 × 10^−16^) (Figure 3F).

**Figure 4:**
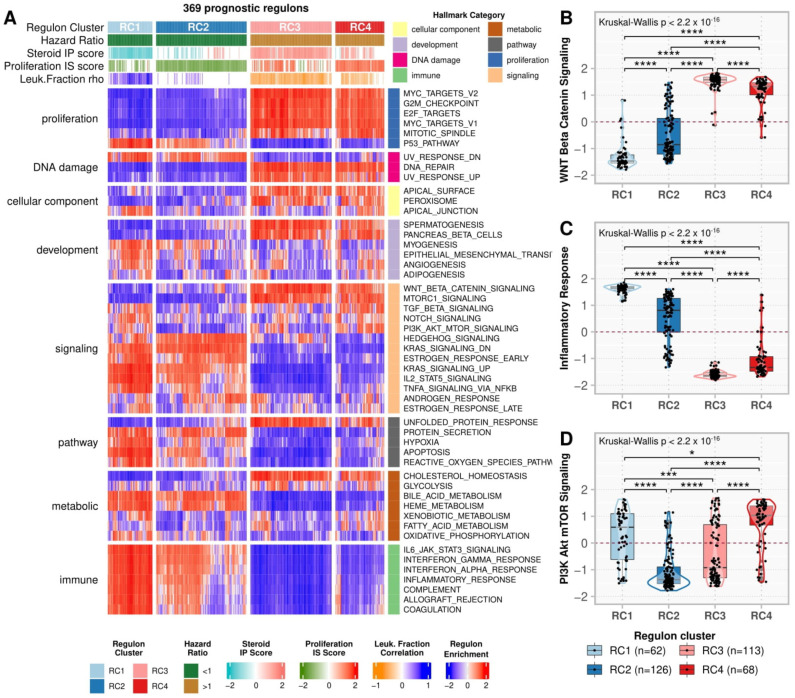
Hallmarks enrichment analysis. (**A**) A heatmap representing the regulon activity enrichment analysis for the MSigDb Hallmarks. Each column represents a prognostic regulon grouped by the regulon cluster, and the rows represent the 50 Hallmarks divided by category. Both rows and columns were subjected to semi-supervised clustering within the groups. In the main heatmap, red indicates a positive enrichment score, while blue indicates the opposite. The top annotation depicts the regulons classification in the clusters, besides the overall survival (OS) hazard ratio (HR), the Steroid independent of Proliferation (IP) and Proliferation independent of Steroid (IS) scores, and the leukocyte fraction correlation for the regulons as presented in Figure 3A. (**B**–**D**) Boxplots comparing the enrichment scores in the regulon clusters for (**B**) Inflammatory Response, (**C**) WNT/β-Catenin Signaling, and (**D**) Mitotic Spindle Hallmarks. Each point represents a regulon separated by the regulon cluster in the x-axis and vertically spread according to its enrichment score for each Hallmark described. The contour presents the distribution density of the regulons for each cluster. The results of Kruskal-Wallis and Dunn’s tests for multiple pairwise comparisons of the ranked data are presented on top. Asterisks indicate the significance level as follows: * *p* ≤ 0.05, *** *p* ≤ 0.001, and **** *p* ≤ 0.0001.

Figure 4A shows the regulon enrichment in the MSigDb Hallmarks (Supplementary Figure S3, Supplementary Table S7). RC3 and RC4, in contrast to RC1 and RC2, were positively enriched in proliferation Hallmarks such as MYC targets v1 and v2, E2F targets, Mitotic Spindle, G2M checkpoint, and WNT/β-Catenin Signaling (Figure 4B). In addition, RC1 was activated, while RC3 was repressed, in the immune Hallmarks (i.e., IL6 JAK STAT3 Signaling, Interferon (IFN) γ and α, and Inflammatory responses—Figure 4C, Complement, Allograft rejection, and Coagulation), in addition to Apoptosis, P53 pathway, and some immune-related signaling pathways (i.e., IL2 STAT5 signaling and TNF-α signaling via NFK-β). On the other hand, RC2 had the lowest, while RC4 had the highest scores for PI3K Akt mTOR (Figure 4D) and Hedgehog signaling.

We also evaluated the RCs concerning the six immune signatures and the TCR metrics inferred by Thorsson et al. (2018) [14] for the TCGA-ACC participants (Supplementary Figure S4A, Supplementary Table S4). RC1 had the highest and RC3 the lowest correlations for Macrophage Regulation and Lymphocyte Infiltrate Signature Scores. Moreover, RC1 and RC2 negatively correlated with Proliferation and Wound Healing signatures, as opposed to the positive values found in RC3 and RC4. The four clusters showed a weak correlation with the IFN-γ Response and TGF-β Response signatures. Regarding the adaptive immune response (Supplementary Figure S4B, Supplementary Table S4), RC1 related positively to TCR Shannon and TCR Richness, while RC3 related negatively to these features. The RC activities did not present a significant correlation with TCR Evenness. Concerning the B cell response, the BCR metrics inferred by Thorsson et al. (2018) [14] were available for only five samples, making comparisons with sufficient statistical power unfeasible.

### 3.3. The ENSAT Cohort Confirmed the Prognostic Value of 89.5% of the Regulons Related to Survival on TCGA-ACC

**Figure 5:**
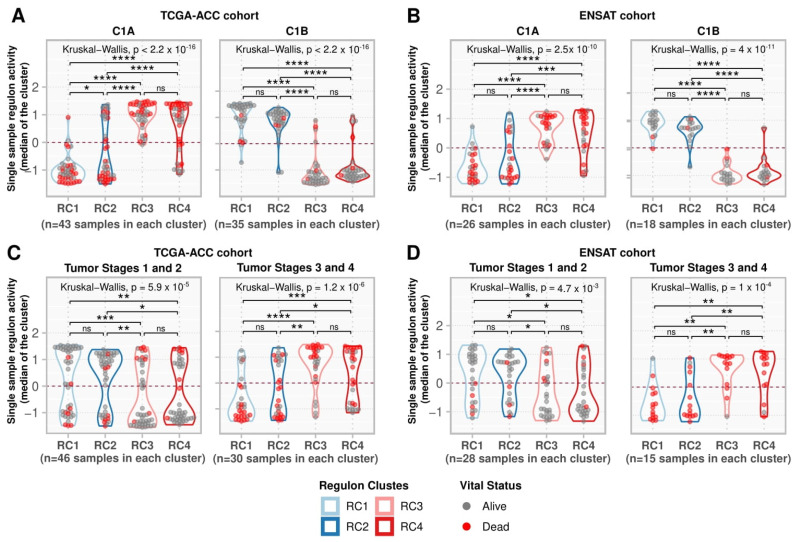
Single-sample regulon activities in regulon clusters. (**A**,**B**) Samples were divided into C1A and C1B as assigned by Zheng et al. (2016) [7] for (**A**) the TCGA-ACC cohort and by Assié et al. (2014) [31] for (**B**) the ENSAT cohort. (**C**,**D**) Samples were divided into a group with tumor stages 1 and 2 and a group with tumor stages 3 and 4 for the (**C**) TCGA-ACC and (**D**) ENSAT cohorts. For each sample, the median regulon activity for each regulon cluster was calculated and represented by the values on the y-axis. Grey points indicate participants alive during the follow-up period, while red indicates participants who died during this time. The contour presents the distribution density of the sample. The results of Kruskal-Wallis and Dunn’s tests for multiple pairwise comparisons of the ranked data are presented on top. Asterisks indicate the significance level as follows: * *p* ≤ 0.05, ** *p* ≤ 0.01, *** *p* ≤ 0.001, and **** *p* ≤ 0.0001. Non-significant *p*-values (*p* > 0.05) are represented by “ns”.

We investigated the regulon activity in the ENSAT cohort (44 ACC samples) (Supplementary Figure S5, Supplementary Table S5) and confirmed the association with survival for the 369 regulons. Of the 369 regulons, 361 were present in the ENSAT gene expression matrix, of which 323 (89.5% of the 361) had significant HRs in multivariate Cox analysis for OS (Supplementary Table S3). High-risk regulons (RC3 and RC4) showed greater activity in the C1A phenotype. They were suppressed in the C1B phenotype, contrary to what was observed for the low-risk regulons (RC1 and RC2), which agrees with the pattern observed in the TCGA-ACC cohort (Figure 5A,B).

Concerning the tumor stages in the TCGA-ACC and ENSAT cohorts, RC1 and RC2 had stronger activity at low tumor stages, while RC3 and RC4 had stronger activity at higher stages (Figure 5C,D). We summarized the results for the 369 regulons in Supplementary Table S4.

### 3.4. NR5A1 Relates to Worse Outcomes in TCGA-ACC and ENSAT Cohorts. CENPA Has a Strong Association with Proliferation and Relates to a Bad Prognosis

**Figure 6:**
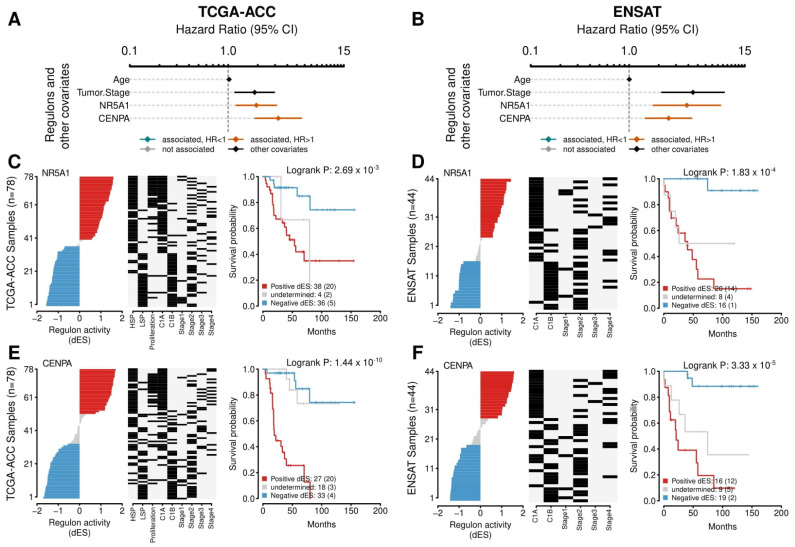
Survival analysis for the *NR5A1* and *CENPA* regulons. (**A**,**B**) A Forest plot with the Hazard Ratios (HRs) and Confidence Interval (CI) for the Cox multivariate analysis. Age and Tumor Stage were used as covariates. The activities of the *NR5A1* and *CENPA* regulons were evaluated for overall survival (OS). (**A**) presents the results for the TCGA-ACC cohort and (**B**) for the ENSAT cohort. (**C**–**F**) The Kaplan-Meier analysis for the *NR5A1* regulon in the (**C**) TCGA and (**D**) ENSAT cohorts and for *CENPA* regulon in the (**E**) TCGA-ACC and (**F**) ENSAT cohorts. In the first and second panels, the rows represent the samples, which were ordered according to the differential enrichment score (dES) for the regulon activity and divided by the median into three groups: positive dES (red), negative dES (blue), and undetermined (grey) as depicted in the first panel. The middle panel shows the molecular classification for tumor stage for each sample, as provided by the publicly available data from the cohorts. The last panel shows the Kaplan-Meier survival analysis between the high and the low regulon activity groups. The adjusted *p*-value for the Log-Rank test is provided. The number of participants in each group is shown, followed by the number of events between parentheses.

*NR5A1* is a well-described TF related to a worse prognosis, adrenal differentiation, and steroidogenesis in ACC. *NR5A1* presented 248 targets in the regulatory network, with 176 negatives and 72 positives (Supplementary Figure S6, Supplementary Tables S1 and S2). In the Cox multivariate analysis, the *NR5A1* regulon related to worse outcomes in PFI (HR = 2.15 [95% CI, 1.49–3.11], *p*-value = 7.12 × 10^−5^) and OS (HR = 1.94 [95% CI, 1.21–3.11], *p*-value = 7.13 × 10^−3^) in the TCGA-ACC cohort, and to OS in the ENSAT cohort (HR = 3.86 [95% CI, 1.61–9.25], *p*-value = 7.45 × 10^−3^) (Supplementary Table S3). The Kaplan-Meier analysis also presented a significant value relating to worse outcomes in OS both in the TCGA-ACC and the ENSAT cohorts (Figure 6A and 6B, respectively).

This regulon presented a significant relation both with HSP (dES = 1.49, *p*-value = 1.65 × 10^−3^) and Proliferation IS phenotypes (dES = 1.33, *p*-value = 1.36 × 10^−3^) and clustered in the RC3. Concerning the immune features analyzed, its activity showed a significant negative correlation with leukocyte fraction (ρ = −0.52, *p*-value = 4.84 × 10^−6^), TGF-β response (ρ = −0.41, *p*-value = 1.71 × 10^−2^, the lowest correlation among the 369 regulons), Macrophage Regulation (ρ = −0.52, *p*-value = 6.84 × 10^−6^), Lymphocyte Infiltration Signature Score (ρ = −0.56, *p*-value = 4.90 × 10^−7^), TCR Shannon (ρ = −0.61, *p*-value = 2.14 × 10^−3^), and TCR Richness (ρ = −0.47, *p*-value = 2.87 × 10^−4^). Moreover, this regulons’ activity showed a significant positive correlation with the Proliferation and Wound Healing signatures (ρ = 0.32, *p*-value = 5.84 × 10^−3^, and ρ = 0.35, *p*-value = 2.07 × 10^−3^, respectively) (Supplementary Table S4).

Within the 369 regulons, we found 150 pairs of regulons that significantly shared targets (Supplementary Table S8) and were therefore called “Duals” [50]. Despite being the second regulon with the most targets (*n* = 248, Supplementary Figure S6), the *NR5A1* regulon did not show significant target sharing with other prognostic regulons.

Of the inferred Duals, Centromeric Protein A (*CENPA*), Small Nuclear RNA Activating Complex Polypeptide 4 (*SNAPC4*), and *LIN54* [a component of the LINC (Linker of Nucleoskeleton and Cytoskeleton) complex, an essential regulator of cell cycle genes], were the regulons with the most associations (9, 8, and 7, respectively) (Supplementary Table S8). Interestingly, *CENPA* had the highest score in the correlation with Proliferation (ρ = 0.90, *p*-value = 4.01 × 10^−27^) and Wound Healing signatures (ρ = 0.79, *p*-value = 2.55 × 10^−15^), and a significant negative correlation with leukocyte fraction (ρ = −0.55, *p*-value = 1.44 × 10^−6^), Macrophage Regulation (ρ = −0.42, *p*-value = 4.29 × 10^−4^), and Lymphocyte Infiltration Signature Score (ρ = −0.48, *p*-value = 2.67 × 10^−5^) (Supplementary Table S4).

We found that *CENPA* was the main regulator of the *MKI-67* gene (MI = 0.77), the most common histopathologic marker of proliferation, followed by *FOXM1* (MI = 0.59), *MXD3* (MI = 0.48), and *DNMT1* (MI = 0.34) (Supplementary Table S1). *MXD3* and *DNMT1* are *CENPA* Duals (Supplementary Table S8), and *FOXM1*, *MXD3*, and *DNMT1* are *CENPA*-positive targets.

**Figure 7:**
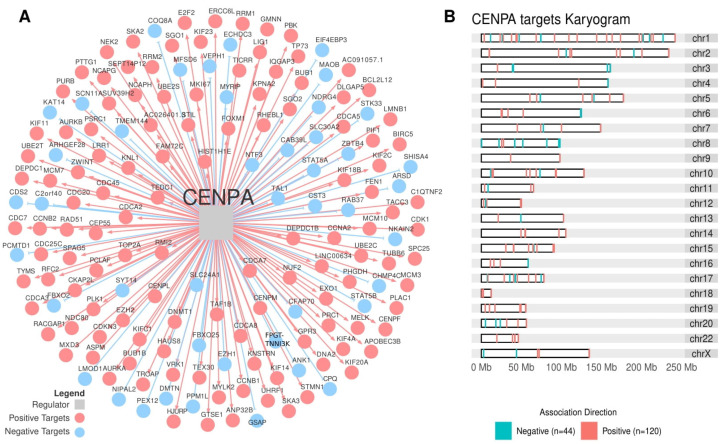
The *CENPA* regulon. (**A**) The transcription factor *CENPA* (grey square at the center) and its targets are inferred by the regulatory network analysis. Blue circles indicate targets with a negative association, while red circles indicate targets with a positive association. (**B**) The karyogram presents the distribution of *CENPA* targets in the chromosomes.

*CENPA* was significantly associated with both the Steroid IP and the Proliferation IS (ρ = 1.45, *p*-value = 1.65 × 10^−3^, and ρ = 1.32, *p*-value = 1.36 × 10^−3^, respectively) and clustered together with RC3 (Supplementary Table S4). In the Cox’s analysis, *CENPA* related to worse outcomes in the TCGA-ACC cohort in PFI (HR = 2.23 [95% CI, 1.54–3.21], *p*-value = 4.55 × 10^−5^) and in OS (HR = 3.24 [95% CI, 1.88–5.57], *p*-value = 1.82 × 10^−4^), in addition to the ENSAT cohort for OS (HR = 2.52 [95% CI, 1.39–4.59], *p*-value = 7.45 × 10^−3^) (Supplementary Table S3). In the Kaplan-Meier analysis, *CENPA* presented a significant relation in the TCGA-ACC and ENSAT cohorts (Figure 6A and B, respectively). Figure 7A presents *CENPA*’s targets, and Figure 7B shows their distribution in a karyogram.

In the TCGA-ACC cohort, the three regulons with higher negative Spearman correlation with *CENPA*’s activity were *THRB* (Thyroid Hormone Receptor Beta)*, STAT5B* (*Signal Transducer and Activator of Transcription 5B*), and *ZBTB4* (Zinc Finger And BTB Domain Containing 4) (ρ = −0.87, −0.86, and −0.85, respectively, all *p*-values < 2.2 × 10^−16^). All of them from RC1 related to good prognosis in the TCGA-ACC and ENSAT cohorts (Supplementary Tables S3 and S4) and presented a negative correlation with the Proliferation signature (ρ = −0.72, −0.75 and −0.68, respectively, all *p*-values < 10^−9^).

## 4. Discussion

The TCGA-ACC cohort is the largest ACC cohort, thus being the most suitable for regulatory network inference. The inferred network had a good balance between positive and negative targets (Supplementary Figure S2), with 611 regulons comprising more than 15 positive and 15 negative targets. We described regulons of prognostic value and provided an overview of the main regulons that control the expression of target genes and, consequently, the observed phenotypes associated with OS. To achieve this, we made functional annotations associating the regulon activity with molecular phenotypes and immunological characteristics.

Here we present 369 regulons associated with OS, where 188 are related to a good prognosis and 181 to a worse ACC prognosis. Within these regulons, we identified subgroups with specific relationships for each set of molecular characteristics and functional annotations, resulting in four RCs. Regulon cluster 1 and RC3 are more related to the Steroid Phenotype, while RC2 and RC4 are more related to the molecular Proliferation Score as defined by Zheng et al. (2016) [7]. As the high Score Proliferation classification was present in 22 of the 47 HSP cases and only 4 of the 31 LSP cases, we distinguished these two features, the Steroid and Proliferation classifications, by analyzing them independently, removing the interference from each other.

RC1 and RC3 are related to Steroid IP, with RC1 associated with LSP and RC3 with HSP. These two clusters presented opposite profiles, both in activity and functional annotations. These clusters were at high activity in the immune and proliferation pathways, as previously shown for the Steroid Phenotype [6]. The strong correlation between RC1 activity and the pro-immune features (Figure 4, Supplementary Figures S3 and S4), in addition to increased activity in early tumor stages (Figure 5B), suggests that regulons in the RC1 may play a role in activating the immune system against different targets at early phases of ACC, which is progressively lost by opposing forces of the RC3 regulons in more advanced stages.

We also observed that RC2 and RC4 follow opposite patterns in terms of activity and characteristics. RC2, with a good prognosis, is related to low scores in the Proliferation IS, while RC4, with a poor prognosis, is related to high scores in this phenotype. We identified from the Hallmarks enrichment (Figure 4, Supplementary Figure S3, Supplementary Table S7) that these prognostic clusters are particularly associated with the PI3K Akt mTOR and Hedgehog signaling pathways, which may help uncover risk factors other than immune response and Steroid Phenotype [5,6,60].

Our functional analysis using the MSigDb Hallmarks presented an overall panorama, providing insights into the biology and function of each group of inferred regulons. However, the MSigDb Hallmarks represent themes of gene sets rather than pathways [30], which may result in supposed contradictory findings. For example, the classical P53 pathway activates DNA repair in response to DNA damage [61,62]. However, in our analysis, the P53 pathway and the DNA repair Hallmarks presented opposite activities (i.e., low for P53 and high for DNA repair in both RC3 and RC4). Increased activity of DNA repair is observed after increased proliferation [62,63], which may explain the observed profiles in RC3 and RC4. In addition, some mutations, such as in the *TP53* gene, may alter the P53 pathway and its DNA repair activity [61,64]. The overall pattern of immune response and proliferation pathways was coherent with the good and poor prognosis clusters. Specific functional correlations need further clarification using in vitro and in vivo studies.

The TCGA pan-cancer analysis, including the ACC cohort by Thorsson et al. (2018) [14], shows that TFs regulating immune modulators tend to be shared between different tissue-of-origin malignancies, in contrast to somatic mutations. They also highlight some immune-related TFs shared among the tumors, of which three appeared in our analysis with association to good prognosis in ACC: *FLI1* (Friend Leukemia Virus Integration 1), *STAT5A* (Signal Transducer and Activator of Transcription 5A), and *IKZF1* (Ikaros Family Zinc Finger Protein 1). For example, in our results, *IKZF1* appeared related to good prognosis (HR = 0.61 [95% CI, 0.40–0.94], *p*-value = 2.65 × 10^−2^), and with the strongest association with the LSP (Steroid IP score of −1.76, *p*-value = 1.65 × 10^−3^) and the highest positive correlation with leukocyte fraction (ρ = 0.83, *p*-value = 5.47 × 10^−18^), Macrophage Regulation (ρ = 0.86, *p*-value = 1.12 × 10^−21^), and Lymphocyte Infiltration Signature Score (ρ = 0.83, *p*-value = 1.56 × 10^−18^) among the 369 regulons (Supplementary Table S4). Finding TFs shared between other tumors and ACC with importance to the immune activation may help to optimize clinical and research efforts in this rare carcinoma.

In ACC, the *NR5A1* TF, an important player in adrenal development and ACC tumorigenesis [65,66], generated one of the largest regulons in our analysis with 248 targets. Consistent with studies on the overexpression of this TF relating to a worse outcome and increased steroid metabolism [23,24,67], the *NR5A1* regulon correlated with low OS in the multivariate Cox and Kaplan-Meier analyses in the TCGA-ACC cohort, which was also confirmed in the ENSAT cohort (Figure 6). Interestingly, of the 369 prognostic regulons, the *NR5A1* regulon showed the lowest correlation with the TGF-β response. Although steroid hormones are well-known immunosuppressors [68,69], the pathways by which they dampen the immune response in the ACC microenvironment are not completely understood. Further studies are needed to examine the immune variables directly altered by *NR5A1* overexpression in the context of ACC.

Remarkably, we identified *CENPA* with interesting correlations with many proliferative markers. *CENPA* is a histone H3-like protein involved in centromeric nucleosome formation [70]. In this study, the *CENPA* regulon showed the highest association with the Proliferation signature. In addition, *CENPA* also appears as the main regulator of *MKI67* (Ki-67), a common prognostic and proliferation marker widely used in cancer histopathology [22].

*CENPA* was described as relating to proliferation and prognosis in ovarian cancer [71], being crucial in prostate cancer growth [72], regulating metabolic reprogramming in colon cancer cells leading to its growth [73], and being associated with immune infiltration and prognosis in lung cancer [74]. Notably, *CENPA* overexpression was also associated with proliferation and metastasis in kidney carcinoma by activating the WNT/β-Catenin signaling pathway [75], an important pathway described in the progression of ACC [3]. In our analysis, *CENPA* appeared positively enriched in this pathway (Supplementary Table S7). Further studies may elucidate whether the relationship in renal carcinoma between *CENPA* activity and the WNT/β-Catenin signaling pathway may apply to ACC.

The three regulons with the highest negative correlation with CENPA’s activity were *THRB*, *STAT5B*, and *ZBTB4*, all from RC1 and of good prognosis. Interestingly, *ZBTB4* is known to act as a tumor suppressor in various types of cancers (prostate cancer [76]; glioma [77]; colorectal cancer [78]; breast cancer [79]; Ewing sarcoma [80]), but more importantly, it is being investigated as a promissory anti-cancer druggable target. For example, Kim et al. (2012) [76] showed that Methyl 2-cyano-3,11-dioxo-18β-olean-1,12-dien-30-oate (CDODA-Me) could increase *ZBTB4* expression in vivo and in vitro, having an antitumorigenic activity in prostate cancer. Whether the increased expression of *ZBTB4* could inhibit *CENPA* and its proliferative activity may be an interesting area for future studies.

It should be noted that our study had some limitations. Our selection strategy may have removed some interesting regulons, which are not listed in the 369 prognostic regulons, thereby excluding their possible role in the ACC regulatory scenario. For example, *FOXM1* is assigned as a prognostic marker in ACC by Yuan et al. (2018) [81]. This TF presented 111 targets, of which only 11 were downregulated, thus not passing through our filter of at least 15 positive and 15 negative targets. We also used protein-coding genes, excluding possible important regulators, such as miRNAs or lncRNAs. Despite these limitations, our results may offer a reference for future studies aiming to understand transcriptional alterations in ACC, prognostic markers, or therapeutic targets.

## 5. Conclusions

In conclusion, we have generated a regulatory network for ACC, evaluated the regulons inferred in concern to OS, clustered them in four RCs, and investigated how they relate to characteristics associated with worse outcomes like the steroid phenotype and the immune and proliferation pathways. The list of prognostic regulons, and their characterization, may open new research avenues and relevant questions to be answered in this hard-to-treat and aggressive malignancy.

## Data Availability

All data and software used in this study are publicly available in the sources described in the Section 2. All scripts and R data (rtni object) to generate results, figures and tables for this study are available on the GitHub repository (https://github.com/sysbiolab/Sup_Material_Muzzi2022) (accessed on 30 August 2022).

## Supplementary Materials

The following supporting information can be downloaded at: https://www.mdpi.com/article/10.3390/cancers14215279/s1, reference [14,30,31]. Figure S1: Distribution of the positive and negative targets of the regulons; Figure S2: Consensus matrix and silhouettes of regulon clusters; Figure S3: Boxplots comparing the Hallmark enrichment scores in the regulon clusters; Figure S4: Boxplots comparing the Spearman’s correlation between the immune features described by Thorsson et al. (2018) [14] and the regulon activity in the clusters; Figure S5: A heatmap showing regulon activity in the ENSAT cohort (*n* = 44 ACC samples); Figure S6: Targets of the *NR5A1* regulon and the related karyogram; Table S1: ACC regulatory network presenting the mutual information between the 1605 transcription factors and their targets; Table S2: Number of positive and negative targets for each regulon; Table S3: Results for the multivariate Cox analysis for overall survival in the TCGA-ACC and ENSAT cohorts, and PFI in the TCGA-ACC cohort; Table S4: A master table resuming the data generated for the 369 prognostic regulons; Table S5: Activity of the 369 prognostic regulons in each sample for the TCGA-ACC and ENSAT cohorts; Table S6: Master Regulator Analysis (MRA) and two-tails gene set enrichment analysis (GSEA2) for the enrichment between the 369 regulons and the Steroid and Proliferation Phenotypes; Table S7: Regulon activity in MsigDb Hallmarks; Table S8: Duals correlation.
