# Supplementary material for: Comprehensive Characterization of the Regulatory Landscape of Adrenocortical Carcinoma: Novel Transcription Factors and Targets Associated with Prognosis
Source: Cancers (Basel). 2022 Oct 27;14(21):5279. doi: 10.3390/cancers14215279 (PMC9657296; doi:10.3390/cancers14215279)
Supplement: Supplementary file 1 [file cancers-14-05279-s001.zip › cancers-1889206-supplementary figures.pdf]

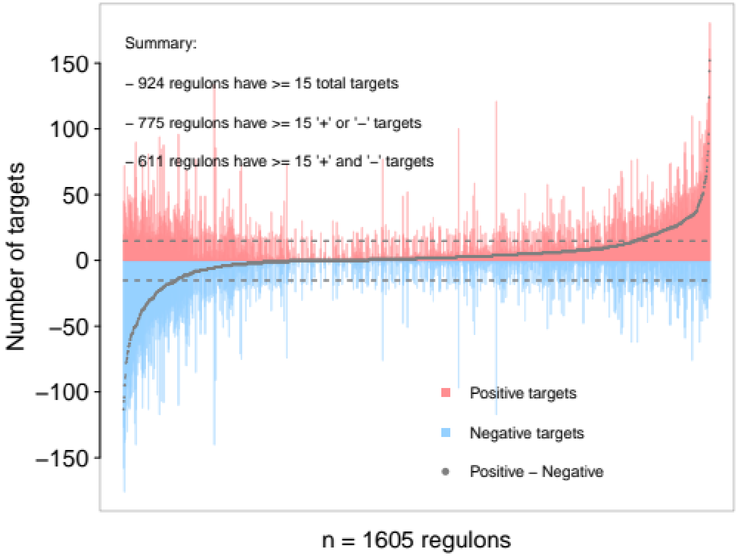

Figure S1. Distribution of the positive and negative targets of the regulons. The 1,605 regulons ordered by the difference between positive and negative targets are presented on the x-axis. Salmon bars show the number of positive targets and blue bars the number of negative targets for each regulon. The dotted lines represent the threshold of 15 targets, the minimum required for gene set enrichment analysis [1].

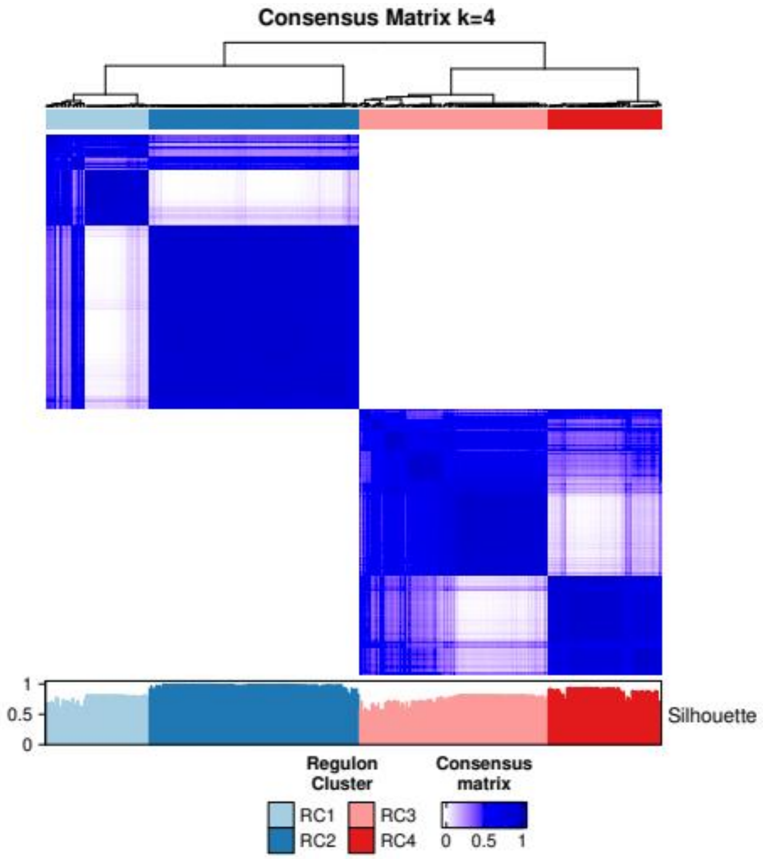

Figure S2. Consensus matrix and silhouettes of regulon clusters. The regulon activity profile was used for the consensus clustering. The consensus matrix for k = 4 is shown in the main heatmap, and the silhouette for each regulon is presented in the barplot below. Regulons are colored according to their respective cluster.

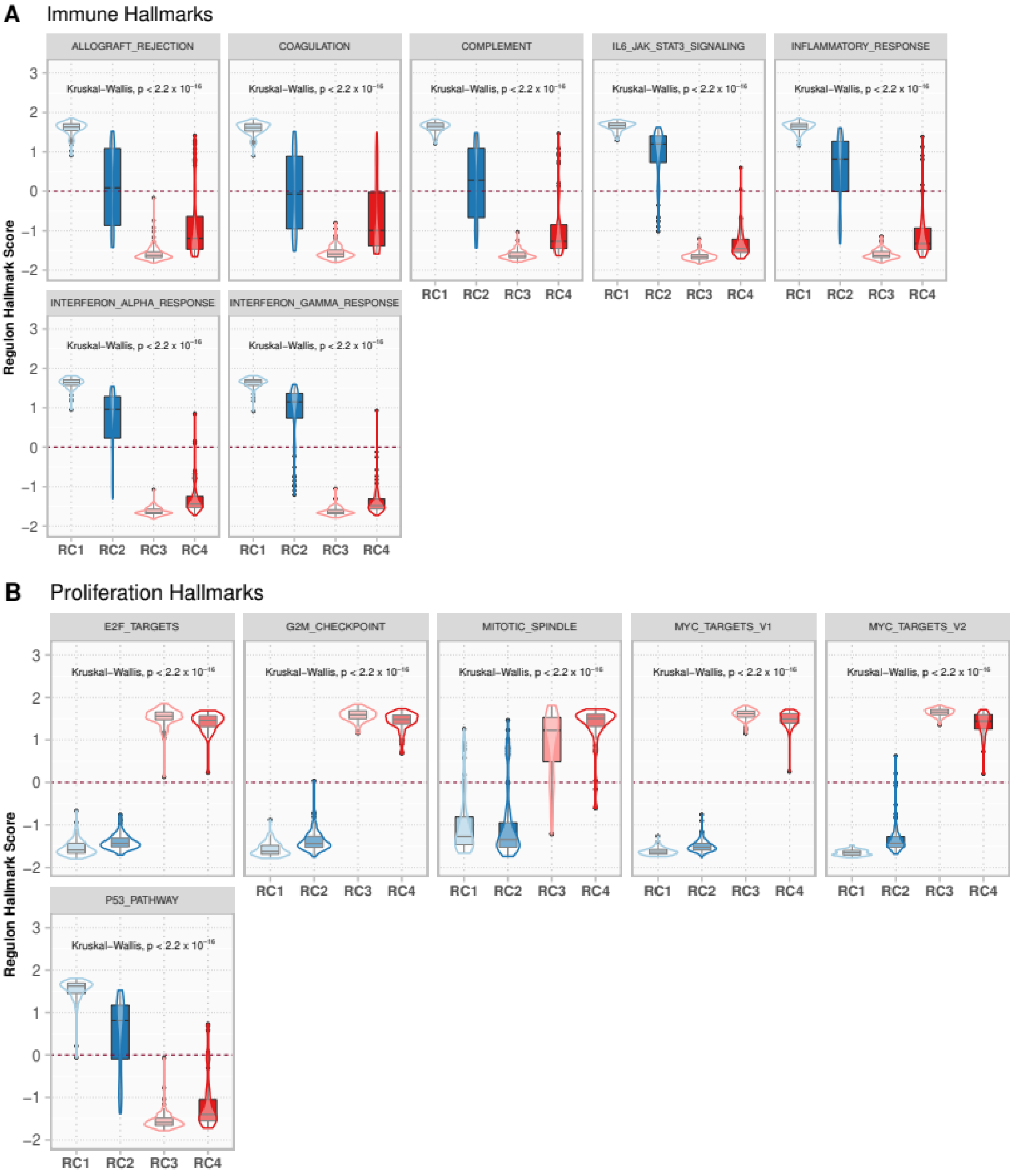
A Immune Hallmarks
ALLOGRAFT_REJECTION
COAGULATION
COMPLEMENT
IL6_JAK_STAT3_SIGNALING
INFLAMMATORY_RESPONSE
INTERFERON_ALPHA_RESPONSE
INTERFERON_GAMMA_RESPONSE
Kruskal-Wallis, p < 2.2 x 10^-16
Regulon Hallmark Score
RC1 RC2 RC3 RC4
B Proliferation Hallmarks
E2F_TARGETS
G2M_CHECKPOINT
MITOTIC_SPINDLE
MYC_TARGETS_V1
MYC_TARGETS_V2
P53_PATHWAY
Kruskal-Wallis, p < 2.2 x 10^-16
Regulon Hallmark Score
RC1 RC2 RC3 RC4

C Signaling Hallmarks

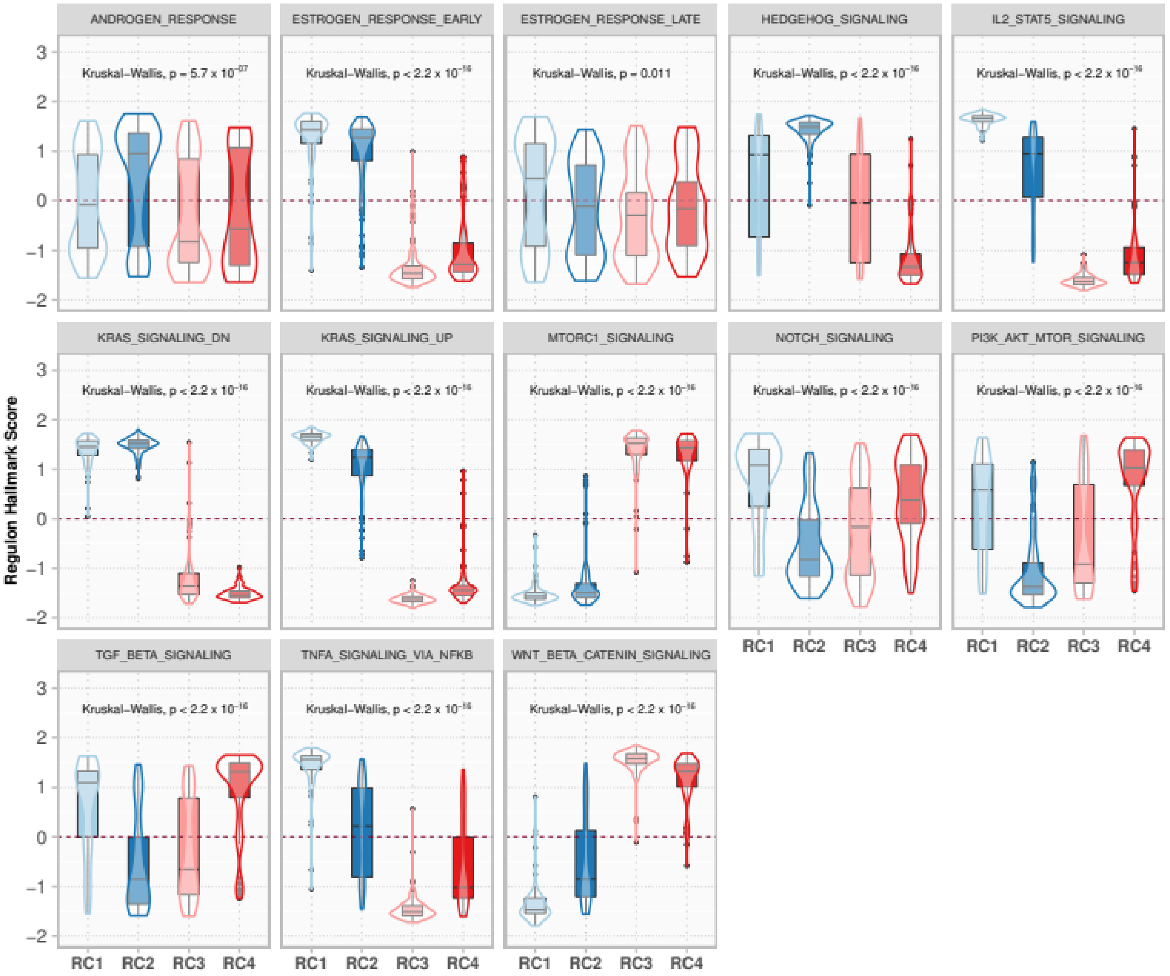

D Pathway Hallmarks

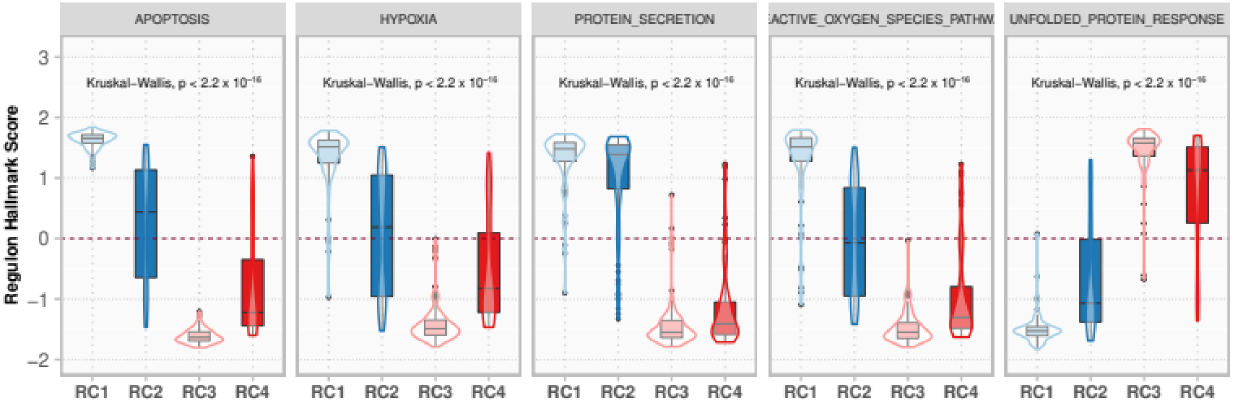

E Metabolic Hallmarks

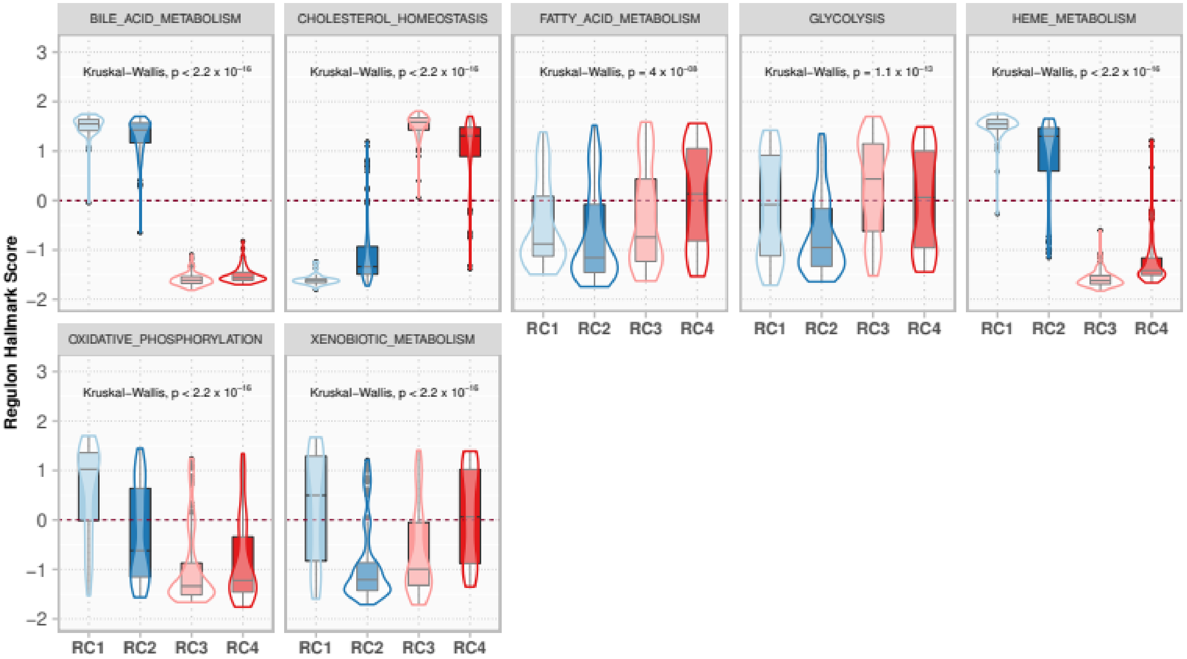

F DNA damage Hallmarks

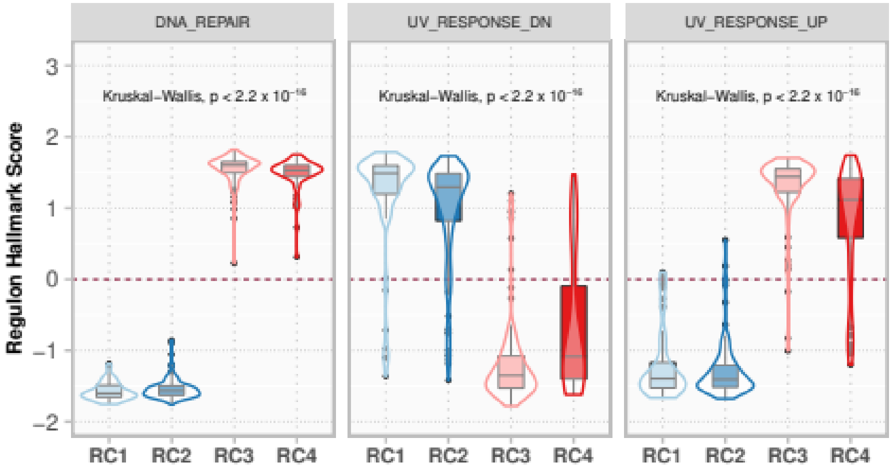

G Cellular component Hallmarks

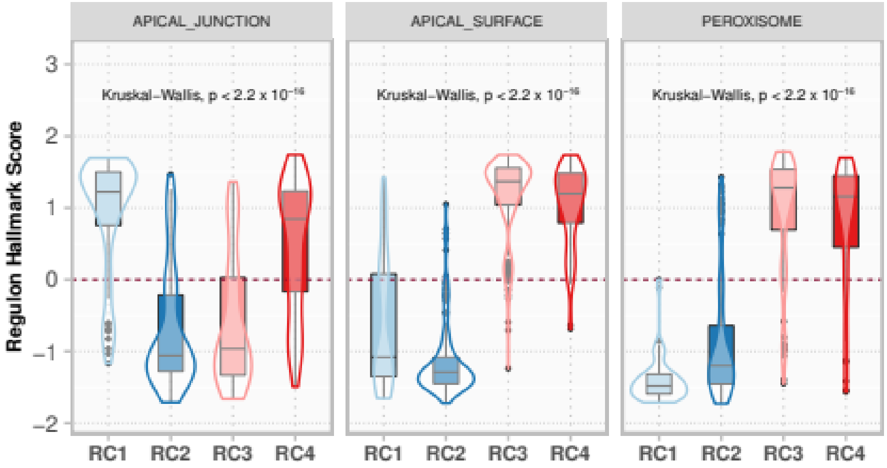

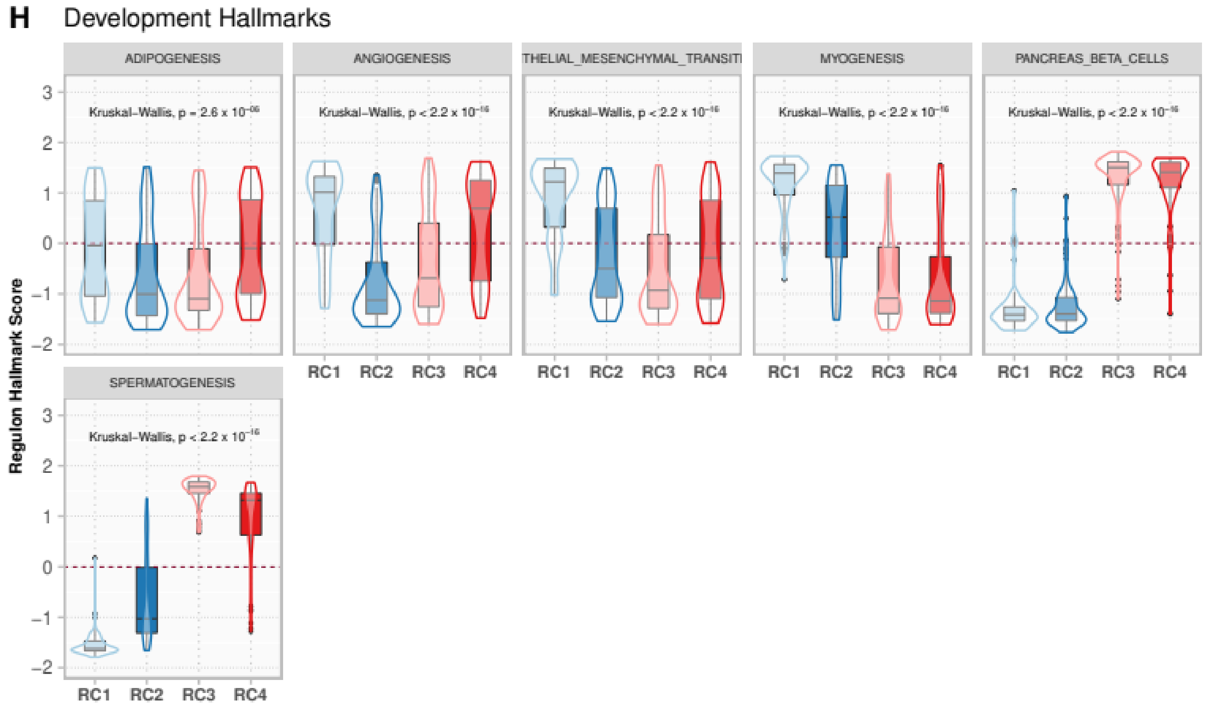

Figure S3. Boxplots comparing the Hallmark enrichment scores in the regulon clusters. The y-axis shows the enrichment score for the regulon in each Hallmark described, while the x-axis separates the regulons in their clusters. The contour presents the distribution density of the regulons for each cluster. The result for the Kruskal-Wallis test is presented on top. The Hallmarks are divided into the following categories: (**A**) Immune, (**B**) Proliferation, (**C**) Signalling, (**D**) Pathway, (**E**) Metabolic, (**F**) DNA damage, (**G**) Cellular component, and (**H**) Development.

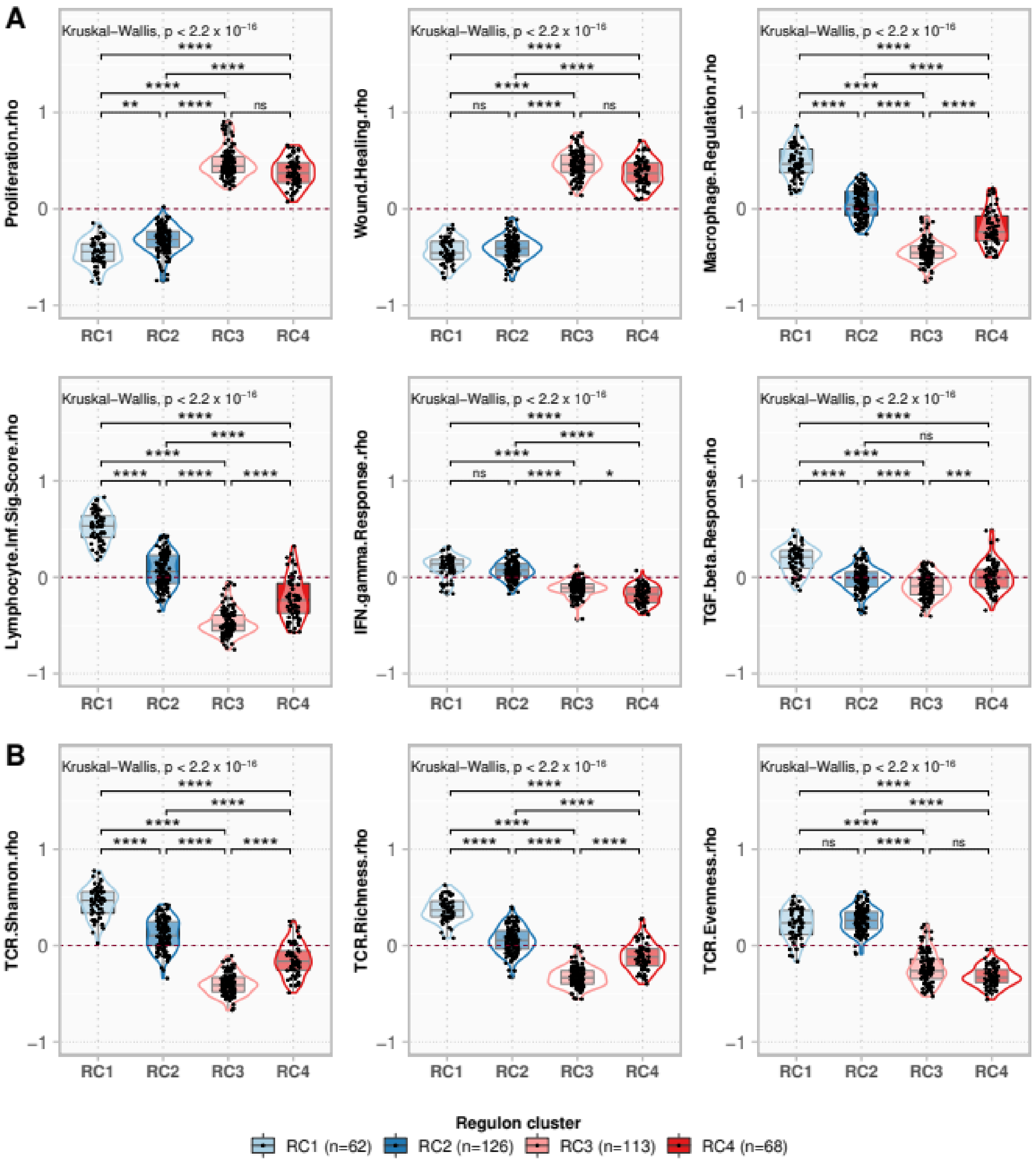

Figure S4. Boxplots comparing Spearman's correlation between the immune features described by Thorsson et al. (2018) [2] and the regulon activity in the clusters. (**A**) Comparison between the correlation for the regulon activity and the six immune signatures score. (**B**) Comparison of the correlation between the regulon activity with the T cell receptor (TCR) metrics. Each point represents a regulon separated by the regulon cluster in the x-axis and vertically spread according to its Spearman correlation to each immune feature. The contour presents the distribution density of the regulons for each cluster. The results of the Kruskal-Wallis and Dunn's tests for pairwise multiple comparisons of the ranked data are presented on top. Asterisks indicate the significance level as follows: *$p \leq 0.05$, **$p \leq 0.01$, ***$p \leq 0.001$, and ****$p \leq 0.0001$. Non-significant p-values ($p > 0.05$) are represented by "ns".

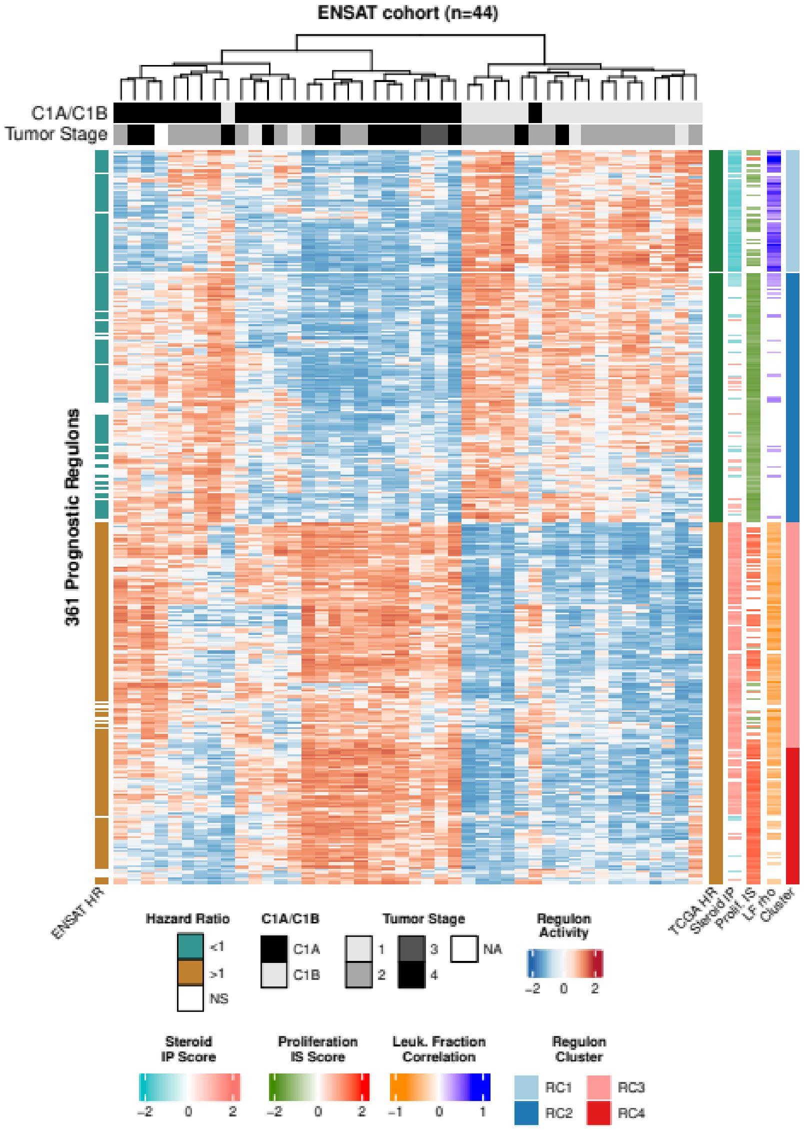

Figure S5. A heatmap showing regulon activity in the ENSAT cohort (n = 44 ACC samples). Each column represents a sample, while the rows represent the 361 prognostic regulons present in the gene expression matrix. The upper track shows the tumor stage and C1A/C1B classification for each sample as described by Assié et al. (2014) [3]. The right track shows the regulon characteristics inferred in the TCGA-ACC cohort, as presented in Figure 3A, while the left track shows the overall survival (OS) hazard ratio (HR) calculated for the ENSAT cohort. .

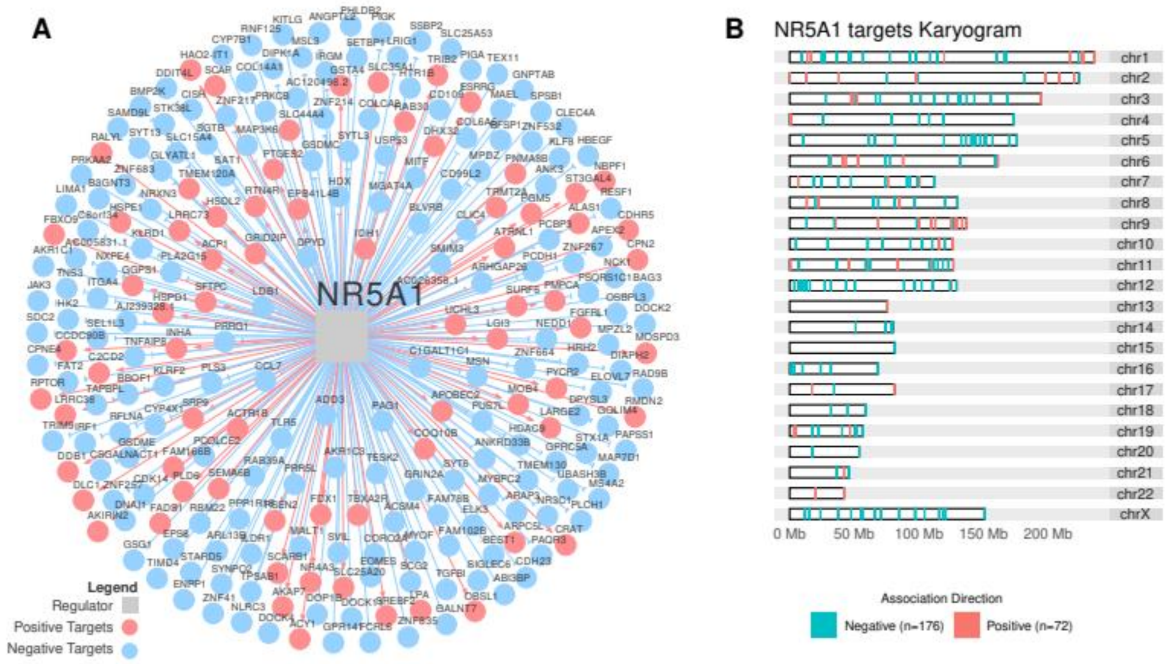

Figure S6. Targets of the *NR5A1* regulon and the related karyogram. (**A**) The transcription factor *NR5A1* (grey square at the center) and its targets inferred by the regulatory network analysis. Blue circles indicate targets with a negative association, while red circles indicate targets with a positive association. (**B**) The karyogram presents the distribution of *NR5A1* targets in the chromosomes.

Reference

1. Liberzon, A.; Birger, C.; Thorvaldsdóttir, H.; Ghandi, M.; Mesirov, J.P.; Tamayo, P. The Molecular Signatures Database Hallmark Gene Set Collection. *Cell Syst.* **2015**, *1*, 417–425, doi:10.1016/j.cels.2015.12.004.
2. Thorsson, V.; Gibbs, D.L.; Brown, S.D.; Wolf, D.; Bortone, D.S.; Ou Yang, T.-H.; Porta-Pardo, E.; Gao, G.F.; Plaisier, C.L.; Eddy, J.A.; et al. The Immune Landscape of Cancer. *Immunity* **2018**, *48*, 812-830.e14, doi:10.1016/j.immuni.2018.03.023.
3. Assié, G.; Letouzé, E.; Fassnacht, M.; Jouinot, A.; Luscap, W.; Barreau, O.; Omeiri, H.; Rodriguez, S.; Perlemoine, K.; René-Corail, F.; et al. Integrated Genomic Characterization of Adrenocortical Carcinoma. *Nat. Genet.* **2014**, *46*, 607–612, doi:10.1038/ng.2953.
